# Upper Extremity Deep Vein Thrombosis as the Tip of the Iceberg: Case Report and Review of Literature

**DOI:** 10.7759/cureus.33513

**Published:** 2023-01-08

**Authors:** Joana Fontes, Bárbara Sousa, Carlos Rego Gonçalves, Joana Faria Silva, Raquel Afonso

**Affiliations:** 1 Internal Medicine, Unidade Local de Saúde do Alto Minho - Hospital Conde de Bertiandos, Ponte de Lima, PRT; 2 Internal Medicine, Hospital de Santa Luzia, Viana do Castelo, PRT

**Keywords:** unprovoked venous thromboembolism, krukenberg tumor, gastric adenocarcinoma, cancer screening strategy, occult cancer

## Abstract

Unprovoked venous thromboembolism (VTE) may be the earliest manifestation of cancer. According to recent studies, approximately 5% of patients with unprovoked VTE will be diagnosed with cancer within the first year of follow-up. Although screening extensively at the time of VTE diagnosis is attractive for clinicians, current clinical guidance documents suggest only a limited cancer screening strategy. The authors describe a rare case of Krukenberg tumor of the ovary arising from a primary gastric adenocarcinoma whose first sign was an unprovoked venous thrombosis of the upper extremity.

## Introduction

There is a well-established association between venous thromboembolism (VTE) and cancer, and VTE may be the earliest manifestation of cancer. The risk of occult cancer is higher in patients with unprovoked VTE [1]. Previous studies reported a 10% incidence of occult cancer diagnosis in the first year of follow-up [2]. Recent studies have reported a lower incidence (approximately 5%), however, it still represents a four to six-fold increased risk as compared with the incidence reported in the general population of the same age [3-5].

Currently, clinical guidance documents recommend only a limited cancer screening strategy at the time of VTE diagnosis, which include a complete medical history and physical examination, blood work (complete blood count, calcium, urinalysis, and liver function tests), chest x-ray, as well as age and gender-specific cancer screening national recommendations [6,7]. The authors described here a rare case of Krukenberg tumor of the ovary arising from a primary gastric adenocarcinoma whose first sign was venous thrombosis of the upper extremity.

## Case presentation

A 61-year-old previously healthy woman presented to the emergency department with a three-day history of left arm pain and swelling. She denied any provoking factors such as trauma, history of prolonged immobility, surgery, or prior venous thromboembolism. She had her cancer screening updated (mammography and fecal occult blood test) [8]. She denied any significant family history. Her vital signs were normal. Her laboratory results, including complete blood count, renal function, and liver function tests, were within normal limits. Left arm ultrasonography revealed thrombosis of internal jugular and subclavian veins. Computed tomography (CT) pulmonary angiography showed acute pulmonary embolism in the subsegmental branches of the lower lobe pulmonary artery, abdominal and pelvic CT was also performed and reported as normal, and Doppler ultrasonography of lower limbs excluded venous thrombosis. She was initiated on direct oral anticoagulant and referred to the internal medicine physician to complete occult cancer screening.

One month later, she was seen on internal medicine consultation complaining of nausea and epigastralgia and was requested cancer screening tests - upper and lower endoscopy, mammography, and re-evaluation of abdominal and pelvic CT due to gastric complaints in addition to blood analysis with tumor markers.

**Figure 1:**
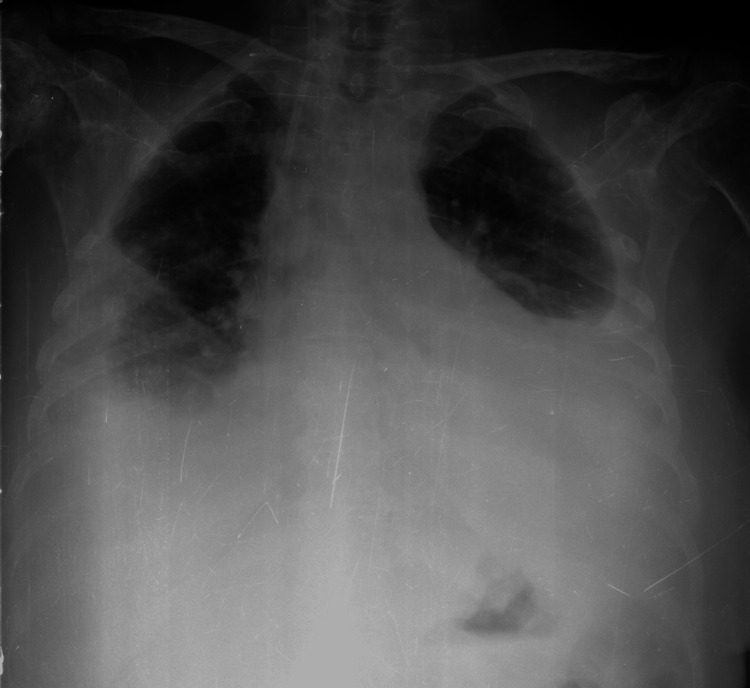
Chest x-ray showing a bilateral pleural effusion.

In the subsequent consultation, two months later, the patient reported new gastrointestinal symptoms, such as anorexia, abdominal discomfort, and vomiting, and complained of exertional dyspnea. She denied blood loss as hematochezia, melena, or hematemesis. Her laboratory results showed elevated alkaline phosphatase (1324 UI/L, normal range: 30-120 UI/L) and serum carbohydrate antigen 125 (CA-125) levels (203 U/mL, normal range: <35 U/mL); the abdominal and pelvic CT revealed periceliac and periaortic lymphadenopathies, signs of mesenteric panniculitis, and an enlarged left ovary (43 mm in diameter). The mammography was normal. At this time, no endoscopy study was yet performed. The patient was electively hospitalized to complete investigation and to treat symptoms of nausea, vomiting, and dyspnea. During the hospitalization, she was diagnosed with large bilateral malignant pleural effusion and required therapeutic thoracocentesis to provide symptomatic relief of dyspnea (Figure 1).

The upper and lower endoscopy was performed and revealed an infiltrative lesion located in the gastric incisura. The anatomopathological evaluation showed a poorly differentiated carcinoma with signet-ring cell features. These signet-ring cells were also identified in the cytology of the pleural fluid.

**Figure 2:**
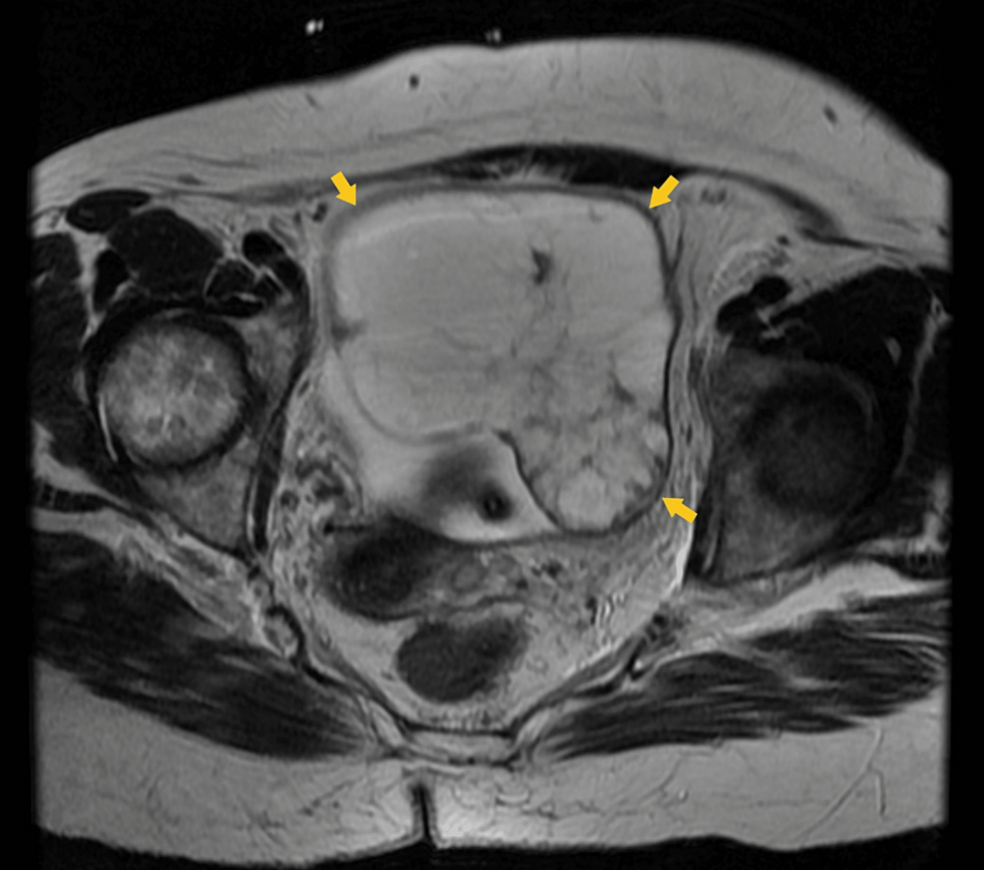
Magnetic resonance imaging of a large ovarian tumor. The axial T2-weighted image demonstrates a left complex cystic mass with solid areas and dimensions of 10.9x8.4 cm (arrows).

Due to the pelvic CT findings, pelvic magnetic resonance imaging (MRI) was performed. It showed a left complex cystic mass with solid areas and dimensions of 10.9x8.4 cm, as well as ascites, metastasis to retroperitoneal lymph nodes, and to spine (Figure 2).

The diagnosis of diffuse metastatic gastric adenocarcinoma was made and Krukenberg tumor of the ovary was presumed. The patient was observed in an oncologic reference center with indication for symptomatic treatment. Unfortunately, the patient experienced rapid deterioration and died seven months after the VTE diagnosis.

## Discussion

Deep vein thrombosis of upper extremity represents 5% of all deep vein thrombosis. It can cause swelling and pain in that extremity and can be complicated by pulmonary embolism. It affects most frequently the subclavian vein but also the jugular, axillary, brachial, or brachiocephalic veins. Most cases are related to underlying risk factors, such as known active cancer or an indwelling central venous catheter, and it rarely occurs spontaneously [6,9].

Recognizing occult cancer as a risk factor for VTE is important, especially when there are no known thromboembolic risk factors, as demonstrated in this case. Several studies have compared limited to extensive cancer screening, especially with the addition of abdominal CT or ^18^F-fluorodeoxyglucose (^18^F-FDG) PET/CT, with the hope to shorten the time to cancer diagnosis and improve cancer-related mortality [10]. To date, they failed to demonstrate its benefit.

In 2015, the Canadian Screening for Occult Malignancy in Patients with Idiopathic Venous Thromboembolism (SOME) randomized controlled trial (n=854) compared a limited occult cancer screening plus CT scanning of the abdomen and pelvis and limited occult cancer screening alone that comprised basic blood testing, chest radiography, and screening for breast, cervical, and prostate cancer. Adding CT did not allow to diagnose more cancers and there was no significant difference between the two study groups in the meantime to a cancer diagnosis or in cancer-related mortality [4].

Robin et al. conducted a multicentre and randomized trial (n=394) in 2015 that evaluated the clinical benefit of adding the ^18^F-FDG PET/CT to a limited screening strategy. The number of occult malignancies detected was not significantly higher in patients in the ^18^F-FDG PET/CT group. However, during a two-year follow-up of individuals with negative initial screening, the risk of subsequent cancer diagnosis was lower in patients who had negative initial screening that included ^18^F-FDG PET/CT. So, it remains to be determined whether ^18^F-FDG PET/CT can be useful in high-risk patients [11].

In 2018, Robin et al. performed a post hoc analysis of a systematic review and meta-analysis of individual patient data of prospective studies that compared limited with extensive screening strategies. They analyzed 1830 patients with unprovoked VTE and conclude that extensive screening was not effective in reducing overall mortality [12].

In this report, the authors described aggressive cancer whose first sign was a venous thrombosis of the upper extremity. According to Portuguese guidelines, for women over 60 years cancer screening with mammography and fecal occult blood test every two years is recommended. At this age, screening for cervical cancer is no longer suggested [8]. Since the patient had these examinations updated, she was initially asymptomatic, had laboratory investigation, and her chest image was normal, it was considered that there was no evidence of cancer at the time of VTE diagnosis. Besides that, an extensive screening with the addition of abdominal and pelvic CT was done and reported as normal. Later, only with the development of symptoms was possible to direct the other screening tests and, finally, to diagnose the tumor.

Krukenberg tumor is a metastatic signet-ring adenocarcinoma of the ovary that primarily arises from the gastrointestinal tract in most cases [13,14]. This is a rare tumor and represents 1-2% of all ovarian tumors [13]. Patients with Krukenberg tumors remain asymptomatic until the disease is very advanced. They usually present with symptoms related to ovarian involvement such as abdominal pain and distension. Since it is a metastatic disease, the prognosis remains extremely poor with a median survival reported in the literature of 16 months [15].

## Conclusions

The diagnosis of an unprovoked VTE should alert physicians to the risk of underlying occult cancer. Although screening extensively is attractive, clinical guidelines only recommend limited cancer screening. In the reported case, at the time of VTE diagnosis, the patient was asymptomatic, and adding the abdominal and pelvic CT didn’t allow to diagnose the tumor earlier nor improve the prognosis.

## References

[1] Gheshmy A, Carrier M (2016). Venous thromboembolism and occult cancer: impact on clinical practice. Thromb Res.

[2] Carrier M, Le Gal G, Wells PS, Fergusson D, Ramsay T, Rodger MA (2008). Systematic review: the Trousseau syndrome revisited: should we screen extensively for cancer in patients with venous thromboembolism?. Ann Intern Med.

[3] Van Doormaal FF, Terpstra W, Van Der Griend R (2011). Is extensive screening for cancer in idiopathic venous thromboembolism warranted?. J Thromb Haemost.

[4] Carrier M, Lazo-Langner A, Shivakumar S (2015). Screening for occult cancer in unprovoked venous thromboembolism. N Engl J Med.

[5] Delluc A, Ianotto JC, Tromeur C (2018). Real-world incidence of cancer following a first unprovoked venous thrombosis: results from the EPIGETBO study. Thromb Res.

[6] Delluc A, Antic D, Lecumberri R, Ay C, Meyer G, Carrier M (2017). Occult cancer screening in patients with venous thromboembolism: guidance from the SSC of the ISTH. J Thromb Haemost.

[7] (2020). Venous thromboembolic diseases: diagnosis, management and thrombophilia testing. http://www.nice.org.uk/guidance/ng158.

[8] (2017). Despacho 2017 criterios rastreios oncolgicos. https://iasaude.pt/attachments/article/3139/despacho_8254_2017_criterios_rastreios_oncolgicos.pdf.

[9] Bosch FT, Nisio MD, Büller HR, van Es N (2020). Diagnostic and therapeutic management of upper extremity deep vein thrombosis. J Clin Med.

[10] Khan F, Vaillancourt C, Carrier M (2017). Should we screen extensively for cancer after unprovoked venous thrombosis?. BMJ.

[11] Robin P, Le Roux PY, Planquette B (2016). Limited screening with versus without 18F-fluorodeoxyglucose PET/CT for occult malignancy in unprovoked venous thromboembolism: an open-label randomised controlled trial. Lancet Oncol.

[12] Robin P, Otten HM, Delluc A, van Es N, Carrier M, Salaün PY, Le Gal G (2018). Effect of occult cancer screening on mortality in patients with unprovoked venous thromboembolism. Thromb Res.

[13] Aziz M, Killeen RB, Kasi A (2022). Krukenberg tumor. StatPearls [Internet].

[14] Lionetti R, DE Luca M, Raffone A (2022). Clinics and pathology of Krukenberg tumor: a systematic review and meta-analysis. Minerva Obstet Gynecol.

[15] Wu F, Zhao X, Mi B (2015). Clinical characteristics and prognostic analysis of Krukenberg tumor. Mol Clin Oncol.

